# Foods for Sleep Improvement: A Review of the Potential and Mechanisms Involved

**DOI:** 10.3390/foods14071080

**Published:** 2025-03-21

**Authors:** Rui Fan, Yingmin Jia, Zhou Chen, Siting Li, Bing Qi, Aijin Ma

**Affiliations:** 1 School of Food and Health, Beijing Technology and Business University, Beijing 100048, China; fr2025220@163.com (R.F.); jiayingmin@btbu.edu.cn (Y.J.); zhouch2017@btbu.edu.cn (Z.C.); lisiting@btbu.edu.cn (S.L.); 2 Hebei Key Laboratory of Walnut Nutritional Function and Processing Technology, Hengshui 053000, China; qibing19870717@126.com

**Keywords:** serotonin, sleep, insomnia, γ-aminobutyric acid, Ziziphi spinosae, lettuce, walnuts

## Abstract

Insomnia affects one-third of the world’s population; the negative effects of insomnia are significant, and traditional insomnia medications have numerous side effects and cause considerable suffering. This has aroused interest in obtaining sleep-improving substances from foods. This study conducted a comprehensive literature review using Web of Science and PubMed with keywords like “sleep”, “insomnia”, and “food”. A subsequent summary of the literature revealed that certain foods, including milk, *Ziziphus jujuba*, *Lactuca sativa*, ginseng, *Schisandra chinensis*, and *Juglans regia*, etc., are purported to enhance sleep quality by prolonging sleep duration, reducing sleep latency, and alleviating anxiety. The mechanisms of these foods’ effects mainly occur via the central nervous system, particularly the gamma-aminobutyric acid (GABA)ergic and 5-hydroxytryptamine (5-HT)ergic systems. Although this review supports the fact that they have potential, further research is needed. There are also issues such as more limited foods, fewer mechanisms, fewer pharmacokinetic studies, and more traditional research models being involved. These need to be addressed in the future to adequately address the problem of insomnia. It is hoped that this study will contribute to research into foods with sleep-improving properties and, in the future, provide an effective natural alternative for those seeking medication.

## 1. Introduction

It has been determined that humans expend one-third of their lives asleep, a period which is critical for the regulation of fundamental physiological processes in both humans and other animal species [1]. Such functions include the maintenance of homeostasis, the development of cortical plasticity, the elimination of neurotoxins, cognitive processes, memory formation, the cultivation of attention and performance, and the regulation of the endocrine and immune systems [2,3]. Sleep can be divided into non-rapid eye movement (NREM) sleep and rapid eye movement (REM) sleep [4]. Each stage of sleep has its own specific function. NREM sleep plays a crucial role in memory consolidation and the clearance of neurotoxins. During this stage, there is an increase in cerebrospinal fluid flow, which accelerates the clearance of neurotoxic substances, such as β-amyloid [5]. Theta waves in the ventral midline thalamus have been shown to initiate memory reactivation and consolidation during sleep [6]. It is imperative to acknowledge the significance of REM sleep in the context of mood, memory, and cognitive function [7]. REM has been demonstrated to play a pivotal role in the elimination of fear and the regulation of anxiety [8,9]. It has been demonstrated that REM sleep facilitates the desensitization of negative emotions [10]. Furthermore, REM sleep projected to the hippocampal CA2 region has been shown to induce the firing of hypothalamic neurons, thereby further consolidating social memory [11].

Sleep is of such significance that it is recommended that adults should obtain a minimum of seven hours of sleep on a regular basis [12]. However, contemporary society is characterized by an alarming prevalence of inadequate sleep, with insomnia currently affecting approximately one-third of the global population [13]. This is mainly due to the fact that there are many factors and mechanisms that lead to insomnia, involving physiological, psychological, and environmental factors [14]. The presence of cancer has been demonstrated to be a contributing factor to the development of insomnia [15]. Furthermore, anxiety and depression have been observed to be associated with insomnia [16]. Aging has been shown to result in NREM sleep disruption [17], and even alcohol consumption has been identified as a potential cause of insomnia [18]. The consequences of insomnia are manifold, including impacts on performance, cognition, and safety, as well as an increased risk of obesity, fatigue, hypertension, diabetes, stroke, Alzheimer’s disease, depression, and other diseases [19,20,21,22]. Although medications such as benzodiazepines, dual orexin receptor antagonists, and melatonin are now available to treat insomnia, the side effects they carry with them, including excessive drowsiness, cognitive impairment, intolerance, headaches, nausea, and rebound insomnia, which can occur after discontinuing the medication, are significant and distressing [23].

Consequently, the development of new substances to improve sleep is of particular importance at this time. In the domain of traditional Chinese medicine, *Ziziphus jujuba*, ginseng, and *Schisandra chinensis* have historically been employed in the treatment of insomnia. Current research has identified a number of foodstuffs, including milk, *Ziziphus jujuba*, *Lactuca sativa*, ginseng, *Schisandra chinensis*, and *Juglans regia*, which have the potential to improve sleep [24,25,26]. The potential benefits of these substances include the capacity to prolong sleep, shorten sleep latency, and alleviate anxiety by modulating GABAergic and 5-HTergic properties. Moreover, they have the capacity to mitigate the adverse effects of insomnia [27]. This has led to a growing interest in the potential of sleep-enhancing substances derived from food sources.

This paper provides a comprehensive overview of the mechanisms of action, sleep-promoting effects, and models for assessing the effects of these foods. A comprehensive evaluation of the extant literature reveals that, while these foods possess the capacity to enhance sleep, the present study encompasses a more restricted array of foods, a reduced number of mechanisms, a paucity of pharmacokinetic studies, and a predominance of traditional research models. It is evident that further in-depth studies are warranted to advance our understanding in this domain. It is hoped that this overview will be useful in the study of foods with sleep-improving properties. It is also hoped that these studies will provide an effective natural alternative in the future for those seeking medication.

## 2. Methods

This comprehensive review sought to provide a thorough overview and analysis of the extant literature on the subject. A comprehensive search was conducted on Web of Science and PubMed using keywords related to sleep disorders and foods that have been shown to improve sleep. The search strategy encompassed the utilization of keywords such as “sleep”, “hypnotic”, “sleep disorders”, “insomnia”, “hypnosis”, “sleep disorders”, “sleep improvement”, “sleep remedies”, “food”, “medicinal food”, and “natural products”. To ensure the inclusion of all relevant studies, no time constraints were applied during the searches. Duplicates were removed, and a total of 120 articles were included in this study. The removal of duplicate and irrelevant literature resulted in the final inclusion of 64 articles in this study.

## 3. Involvement in Sleep Regulation Mechanisms

Sleep can be considered not only as a passive response of organisms to environmental changes but also as an active process occurring within the central nervous system. Sleep is a complex physiological process involving multiple neurotransmitters, hormones and neural pathways. Substances such as GABA, 5-HT, dopamine (DA), norepinephrine (NE), adenosine, prostaglandin D2 (PGD2), cytokines, and melatonin work together to regulate sleep homeostasis and circadian rhythms [28,29,30,31]. Together, these mechanisms regulate sleep induction, maintenance, and arousal. Further research has been conducted on the mechanisms involved in the improvement of natural sleep products, with studies focusing on 5-hydroxytryptamine, melatonin, and gamma-aminobutyric acid.

### 3.1. Gamma-Aminobutyric Acid (GABA)

GABA is a non-protein amino acid that is the major inhibitory neurotransmitter in the nervous system, with actions including the inhibition of neuronal excitation, anti-epileptic, sleep promoting, and stress and anxiety regulating [32,33,34,35]. In normal models, GABA transmits inhibitory signals via receptors that promote deep sleep and improve sleep quality. In normal models, GABA transmits inhibitory signals via receptors that promote deep sleep and improve sleep quality. In disease models, GABA not only induces sleep but also attenuates nociceptive hypersensitivity induced by ovarian hormone withdrawal, reduces amyloid-β deposition, and reverses memory deficits in Alzheimer’s disease models [36,37]. It has been established that flavonoids extracted from mulberry leaves, peptides from milk (YPVEPF and YFYPEL), and jujube extract can play a role in improving sleep by regulating the GABAergic system [24,38,39].

**Figure 1:**
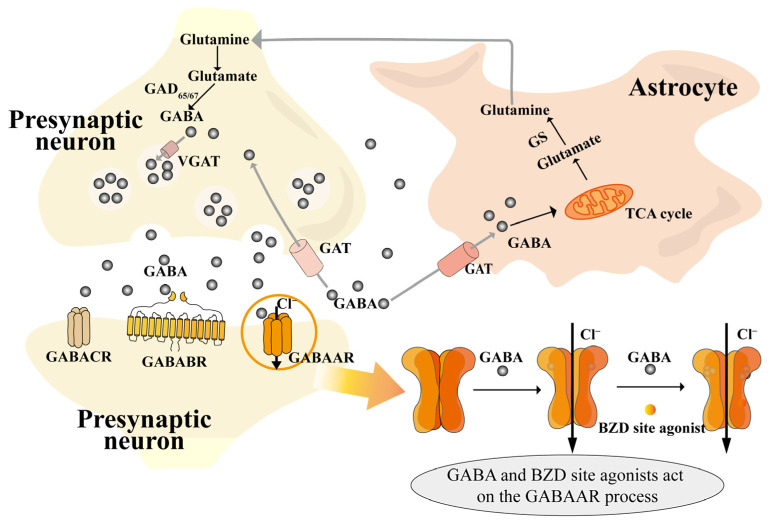
Diagram of GABA mechanism of action [40,41,42,43,48]. Notes: BZD site (benzodiazepine site); GABA (gamma-aminobutyric acid); GABAAR (gamma-aminobutyric acid type A receptor); GABABR (gamma-aminobutyric acid type B receptor); GABACR (gamma-aminobutyric acid type C receptor); GAD_65/67_ (glutamate decarboxylase 65/67); GAT (gamma-aminobutyric acid transporter); GS (glutamine synthetase); TCA cycle (tricarboxylic acid cycle); VGAT (vesicular gamma-aminobutyric acid transporter).

The metabolism, transport, and action of GABA are shown in Figure 1. GABA is synthesized from its precursor, glutamate (Glu), within the cytoplasm of presynaptic neurons by the action of the enzyme glutamate decarboxylase (GAD, including GAD65 and GAD67) [40]. GABA then enters the vesicle via a vesicular transporter protein (VGAT), and GABA is released through the vesicle into the synaptic cleft to participate in the next reaction. Part of it is transported by transporter proteins (GAT, including four types, namely GAT1, GAT2, GAT3, and betaine/GABA transporter 1) to astrocytes for the tricarboxylic acid cycle (TCA) and then for the synthesis of glutamine (Gln) catalyzed by glutamine synthetase (GS) [41,42,43]. Finally, Gln is transferred from astrocytes to neurons via sodium-coupled neutral amino acid transporters (SNATs), where Gln is involved in the next step of GABA synthesis; part of it is transported back to the synapse via GAT, and the other part binds to GABA receptors on postsynaptic neurons to play a role.

GABA is delivered via GABAA receptors (GABAAR) and GABAB receptors (GABABR) and balances excitatory neurotransmitters such as Glu, norepinephrine (NE), 5-HT, acetylcholine, orexin (OX), and dopamine (DA), which are released during sleep and are capable of increasing arousal activity [44]. Of these, GABAAR is the more heavily studied, and the binding of GABA to it opens the chloride channel, which can, for a short period of time (milliseconds), increase anion conductance, leading to the hyperpolarization of depolarized membranes and prevention of action potential transmission [45]. GABAAR [46] receptors are members of the Cys-loop superfamily of ligand-gated ion channels responsible for tachykinetic inhibitory synaptic transmission in the brain. These receptors aggregate as pentamers, with five subunits arranged around a central axis to form an ion-permeable pathway through the plasma membrane. These heteropentameric protein complexes are composed of a combination of eight subunit classes, encoded by nineteen different genes, namely α1-6, β1-3, γ1-3, δ, ε, θ, π, and ρ1-3 [47]. The activation of GABAAR instigates the opening of the intrinsic chloride channel, consequently resulting in chloride influx, the hyperpolarization of the membrane potential, and a decrease in neuronal excitability [48]. This engenders a sedative, hypnotic effect, and it is precisely this effect that is mimicked by hypnotic drugs (barbiturates and benzodiazepines), which are based on GABAA receptor-mediated action. Of particular note are benzodiazepines such as zolpidem and zopiclone, which are coupled to GABAAR and modulated in a metabotropic manner. This enhances the affinity of GABA for its receptor, thereby augmenting its effect [45].

### 3.2. Tryptophan (Trp)

Trp is an essential amino acid involved in a variety of physiological processes in living organisms, such as the synthesis of neurotransmitters (5-HT and melatonin (MT)). There are three main metabolic pathways for tryptophan in the human body: the kynurenine pathway, the indole pathway, and the pathway for the synthesis of 5-HT and MT [49]. Of these, the pentraxin and melatonin synthesis pathways are very important in the regulation of sleep.

**Figure 2:**
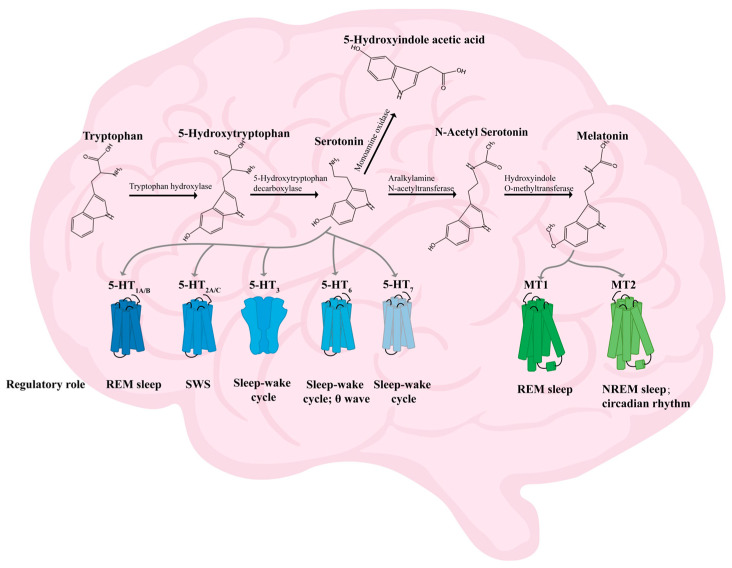
Diagram of the mechanism of action of 5-HT and MT [50,51,52,53,58,59,60,61,62,63,71]. Notes: MT1 (melatonin receptor 1); MT2 (melatonin receptor 2); NREM sleep (non-rapid eye movement sleep); REM sleep (rapid eye movement sleep); SWS (slow-wave sleep); 5-HT_1A/B_ (5-hydroxytryptamine receptor 1A/B); 5-HT2_A/B_ (5-hydroxytryptamine receptor 2A/B); 5-HT_3_ (5-hydroxytryptamine receptor 3); 5-HT_6_ (5-hydroxytryptamine receptor 6); 5-HT_7_ (5-hydroxytryptamine receptor 7).

Trp is the only precursor for the production of 5-HT in both the peripheral and central nervous systems. Less than 5% of this is synthesized as 5-hydroxytryptophan(5-HTP) via TPH, which further generates 5-HT via 5-hydroxytryptophan decarboxylase and pyridoxal cofactor enzymes [50]. 5-HT can be catabolized to 5-hydroxyindole aldehyde (5-HIA) via monoamine oxidase (MAO), and 5-HIA can then be metabolized to 5-hydroxyindoleacetic acid (5-HIAA) by aldehyde dehydrogenase (ALDH) [50,51]. 5-HT is also a precursor for the synthesis of the MT, which is synthesized as N-acetyl-5-hydroxytryptamine (NAS) via aralkylamine N-acetyltransferase (AANAT), and then melatonin is produced by hydroxyindole O-methyltransferase [52,53] (Figure 2).

5-HT is an indole derivative that was first identified in blood serum. It is also referred to as serotonin. This neurotransmitter plays a crucial role in various physiological processes, including sleep regulation, body temperature control, immune function, mood stabilization, and cognitive function [54,55,56]. The receptor type of 5-HT is complex and is now classified into seven categories based on structural and action characteristics (5-HT_1_-5-HT_7_) [57]. The 5-HT_1A/B_ receptors are involved in the regulation of REM sleep [58]; the 5-HT_2A/C_ receptors are involved in the modulation of wakefulness and slow-wave sleep (SWS) [59,60]; the 5-HT_6_ receptors are involved in regulating the sleep–wake cycle and theta wave [61]; and both 5-HT_3_ and 5-HT_7_ are involved in the sleep–wake cycle and REM sleep regulation [62,63]. The prevailing opinion is that 5-HT belongs to the category of inhibitory neurotransmitters [57]. For instance, the classical insomnia model drug *p*-chlorophenylalanine (PCPA) exerts an inhibitory effect on tryptophan hydroxylase (TPH) [64], the rate-limiting enzyme for the synthesis of 5-HT. Modified Suanzaoren Decoction (MSZRD) has been demonstrated to enhance sleep quality by modulating neurotransmitter levels, including 5-HT, NE, DA, GABA, and Glu. This regulation involves the reduction in OX-A and OX2 expression, thereby restoring hypothalamic–pituitary–adrenal (HPA) axis homeostasis [65]. However, 5-HT has been observed to promote arousal after sleep onset. The precise function of 5-HT in sleep remains to be fully elucidated, given its divergent effects in different states and its multifaceted actions on various receptors. However, it is widely acknowledged that 5-HT plays an indispensable role in sleep.

5-methoxy-N-acetyltryptamine (melatonin; MT) has been demonstrated to possess sleep-regulating, immune response-promoting, and anti-tumor properties [66,67,68]. The synthesis of melatonin occurs in two distinct locations, namely the pineal gland and the retina, specifically in pineal cells and photoreceptors, respectively [69]. It has been observed that the synthesis of melatonin is reduced during daytime hours and elevated at night [70]. Melatonin exerts its effects through the activation of melatonin receptor 1 (MT1) and melatonin receptor 2 (MT2). MT1 is involved in regulating REM sleep, while MT2 is involved in regulating NREM sleep and circadian rhythms [71]. Yi [72] et al. showed that soy peptides prolong sleep time by increasing the expression of TPH, AANAT, MT1, and MT2 in the mouse brain and promoting the conversion of the neurotransmitter 5-HT to MT.

## 4. Foods with Sleep-Improving Properties

**Figure 3:**
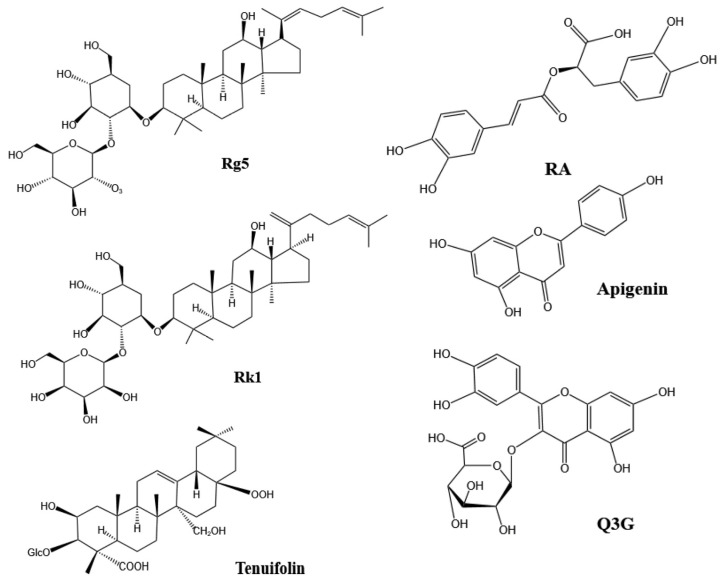
Structure of active ingredients for sleep improvement [110,122,150,151,152] Notes: RA (Rosmarinic acid); Rg5 (Ginsenoside Rg5); Rk1 (Ginsenoside Rk5); Q3G (Quercetin-3-glucuronide).

There are drugs available to treat insomnia; they work quickly but have more serious and painful side effects [23]. And drugs like benzodiazepines are recommended for short-term use, with a maximum duration of no more than four weeks [73]. So, research into new substances to improve sleep is particularly important during this time. Current research shows that herbal supplements are relatively safe and well tolerated, with few adverse effects [23]. And substances such as milk and ginseng have been reported to have the potential not only to improve sleep, but also to reduce the side effects associated with insomnia [74,75]. Therefore, extracting sleep-improving substances from food is very promising. Components, models, and their mechanisms of action that have the potential to improve sleep from food are shown in Table 1. The structure of the active ingredients for sleep improvement is shown in Figure 3.

### 4.1. Milk

Milk has the potential to improve sleep [76]. A comprehensive review of the literature on the effects of milk and dairy products on sleep found that the ingestion of milk and dairy products was associated with improved sleep [77].

Research has shown that active peptides from milk improve sleep. Bovine milk casein trypsin hydrolysate has been demonstrated to possess sleep-improving and anxiolytic properties [78,79]. Miclo [80] et al. examined the affinity of peptides isolated from its hydrolysate for the GABAAR, and of these, only the 91-100 (YLGYLEQLLR) fragment of the αs1-casein was expressed to have affinity for the GABAAR. Qian [24] et al. Consequently, it was observed that bovine milk casein trypsin hydrolysate significantly prolonged the sleep time of mice, reaching a nearly twofold increase compared to the decapeptide YLGYLEQLLR identified in a previous study. Moreover, two peptides, YPVEPF and YFYPEL, which exhibited potent sleep-promoting activity, were subjected to virtual screening to identify their potential docking with the GABAAR. Subsequently, Chen [81] et al. established an in vitro model based on an electrophysiological model of brain neurons to assess the sleep-promoting effects. Based on the previous studies, four novel peptides were isolated from bovine casein tryptic hydrolysate: HQGLPQEVLNENLLR (αs1-CN, f8-22), YKVPQLEIVPNSAEER (αs1-CN, f104-119), HPIKHQGLPQEVLNENLLR (αs1-CN, f4-22), and VPQLEIVPNSAEER (αs1-CN, f106-119). Subsequent investigation by Qian [82] revealed that casein hydrolysates enriched with YPVEPF peptides could reverse the behavioral consequences of chronic stress, including anxiety and insomnia, by modulating the anabolism of neurotransmitters such as GABA and 5-HT. This modulation also encompassed the HPA response, the ERK/CREB-BDNF-TrκB signaling pathway, and the attenuation of inflammation in mice.

Research has shown that dairy products also have the potential to improve sleep. Yamamura [83] et al. found that 60–81-year-olds who ingested Lactobacillus helveticus fermented milk showed a significant increase in sleep rate, a significant decrease in the number of awakenings, and a tendency to decrease sleep latency. Yu [84] et al. found that the use of a high-producing strain of GABA Lactobacillus brevis DL1-11 fermented milk and administered it to ICR mice, in which the high-dose group exhibited reduced anxious behavior, augmented sleep duration, and diminished sleep latency. The relative abundance of *Ruminococcus, Adlercreutzia,* and *Allobaculum* in the high-dose group, as well as the levels of some short-chain fatty acids (SCFAs), were found to be significantly increased. The results showed that the sleep-improving effect of the fermented milk might be related to the regulation of the intestinal flora and the enhancement of SCFA levels.

Overall, certain components of milk and dairy products, as well as the consumption of fermented dairy products, have the potential to promote sleep, particularly by regulating the balance of neurotransmitters and gut flora.

### 4.2. Ziziphus jujuba

*Ziziphus jujuba* Mill. *var. spinosa* is native to China, and its fruits, seeds, leaves, and other parts can be used as food, medicine, and health care products [85]. In Chinese medicine, sour jujube seed is used as the main treatment for restlessness and insomnia, sweating and thirst deficiency, and soreness of limbs. It is rich in terpenoids, flavonoids, saponins, alkaloids, anthocyanins, polysaccharides, lignans, and other active ingredients [86,87,88,89,90]. Recent studies have found many functions: hepatoprotective, antioxidant, sedative, hypnotic, and anxiolytic functions, treatment of depression, neuroprotective effect, improvement of memory, and cognitive ability, etc. [91,92,93,94]. Additionally, the EtOH–water extract of *Ziziphus jujuba* leaves displays a hepatoprotective function [95].

Ziziphi Spinosae Semen (ZSS) is the drug of choice for the treatment of insomnia in traditional Chinese medicine, with a history of clinical use spanning many years. It has now been demonstrated that ZSS and its extracts can improve sleep and alleviate the effects of insomnia on the body through a wide range of pathways, including regulating the expression of neurotransmitters, improving energy metabolism levels, correcting the dysfunction of the HPA axis, enhancing immune function, and regulating intestinal flora disorders [96].

Complex extracts from ZSS have been shown to improve sleep. Sun [97] et al. found that essential oils extracted from ZSS were able to increase sleep rate, decrease sleep latency, and increase sleep duration in mice. This was achieved through modulation, mainly by the up-regulation of 5-HT, GABA, and GABAAR and the downregulation of Glu and NE. Hua [98] et al. treated PCPA-modeled sleep deprivation (SD) rats with ZSS extracts and found that it improved insomnia. The analysis of plasma and urinary metabolites using 16S rRNA, in conjunction with gut flora studies, revealed that ZSS could improve insomnia by regulating amino acid metabolism disorders and reversing insomnia-induced disturbances and SCFA abnormalities. Hua [99] et al. found that the ethanolic extract of ZSS could play an anti-insomnia role by significantly reducing the serum levels of corticotropin-releasing hormone (CRH), adrenocorticotropic hormone (ACTH), interleukin-1β (IL-1β), and tumor necrosis factor-α (TNF-α) in insomniac rats and by regulating the secretion of various hormones from the hypothalamus to correct the disorder of the HPA axis.

Saponins and jujubosides extracted from ZSS have been shown to improve sleep. Cao [100] et al. found that jujubosides extracted from ZSS could significantly increase total sleep and REM during the day (9:00–15:00) and total sleep and NREM during the night (21:00–3:00) in mice. They also significantly enhanced the hypnotic effect of sodium pentobarbital. Further analysis revealed that the hypnotic effect of jujubosides may be achieved through the modulation of circadian rhythms and the 5-HT system. And the saponin jujuboside A (JuA) isolated from ZSS is the main active ingredient [101]. Wang [102] et al. stimulated rat hippocampal neurons using rat small intestine tissue cultures treated in vitro and later found that JuA affects the interneuronal cellular neuronal network in the brain by modulating the expression of mRNA of the GABA receptor subunit and down-regulating the secretion of inflammatory molecules associated with the intestinal mucosal system. It exerts a special sedative and hypnotic effect. Subsequently, Song [103] et al. investigated the gastrointestinal absorption and metabolic kinetics of JuA, determining that its utilization was 1.32% in rats. The poor bioavailability of JuA was primarily attributable to metabolic processes, with its metabolites, jujuboside B (JuB) and jujubogenin, exerting a significant effect on the expression and activation of GABAR, indicating that the metabolites of saponins are responsible for specific biological activities. Sanjoinine A, one of the major alkaloid compounds in ZSS, was found by Ma [104] et al. to enhance sodium pentobarbital-induced sleep behavior by altering the GABAergic system, prolonging pentobarbital-induced sleep duration, and reducing sleep latency in a dose-dependent manner. Wang et al. then conducted a study using co-administration (including jujuboside B and spinosin) and found that co-administration was more effective than single administration. It resulted in mice with longer sleep durations and shorter sleep latency.

In summary, ZSS has many advantages in improving sleep. These include the high number of active ingredients in ZSS, the wide range of mechanisms involved, and the low incidence of adverse events compared to sleeping pills [105]. This makes it ideally suited as an ingredient for sleep improvement products or drug development.

### 4.3. Lactuca sativa

*Lactuca sativa* (lettuce), scientifically known as *L. sativa* L., is a vegetable in the Asteraceae family that is widely grown throughout the world. It is considered a nutrient-rich vegetable due to its high water content and richness in dietary fiber, B vitamins, glycosylated flavonoids, and sesquiterpene lactones [106]. In addition to being consumed as a food, it improves insomnia and lowers blood lipids, oxidative damage, cancer, and neurodegenerative diseases [26,107,108].

At the animal level, lettuce extract has been shown to improve sleep. Ghorbani [109] et al. investigated the sleep-improving activity of the hydroalcoholic extract (HAE), water fraction (WF), ethyl acetate fraction (EAF), and n-butanol fraction (NBF) of lettuce and found that only the HAE and NBF fractions were able to prolong sleep time in mice. Subsequently, ethanol-extracted green lettuce leaves were shown to affect sleep behavior in both invertebrate and vertebrate models of physical stress-induced sleep. These extracts were able to restore sleep lost during vibratory stress in *Drosophila melanogaster* and to improve stress-induced sleep pattern changes in rats by modulating GABAAR and increasing total sleep time, NREM, and delta waves [110]. The sleep-promoting effects of romaine lettuce extract in rodents were also found to be mediated by GABAergic mechanisms by Kim et al. [111] The aqueous extract of Heukharang lettuce leaf was able to increase total sleep time, NREM, and delta waves in a sodium pentobarbital-induced rodent sleep model and to reduce wake time in a caffeine-induced insomnia model. Further investigation revealed that it exerts its sleep-improving effects by modulating benzodiazepine receptors at the GABAAR [26]. The extract from Heukharang was also able to participate in sleep promotion by modulating adenosine receptors and had no adverse effect on spontaneous motor activity and motor coordination in mice compared to diazepam [112].

At the human level, lettuce seed oil has been shown to improve sleep. Yakoot [113] et al. found that lettuce seed oil improved sleep difficulties in elderly insomniacs with and without anxiety, with no side effects. Another clinical trial found that lettuce seed oil improved sleep quality in pregnant women aged 20–45 with insomnia [114].

In summary, the mechanism of action of lettuce in regulating sleep is mainly related to GABA, and it does not exert the same side effects caused by traditional sleeping pills in rodents. Additionally, it has been shown to improve sleep in special populations such as the elderly and pregnant women, making it a promising substance for sleep improvement.

### 4.4. Ginseng

In East Asian countries, ginseng has been utilized as both an herbal remedy and a functional food over the past few decades. It has been demonstrated to possess a range of pharmacological functions, including anti-inflammatory and immunomodulatory effects, cardiovascular disease management, the treatment and prevention of insomnia, antidepressive effects, and cognitive enhancement properties [74,115].

Ginseng ethanol extract (GEE) has been shown to improve sleep. GEE was found to regulate the sleep structure of rats, reducing the number of sleep–wake cycles and increasing the duration of NREM sleep [116]. GEE promotes orexin-mediated autophagy to exert functions such as prolonging sleep duration, improving cognitive function, and preventing hippocampal neuronal damage in aged sleep-deprived rats [74].

The saponins in ginseng have been shown to improve sleep. According to recent studies, ginseng’s functional activities are often mediated by its saponins [117,118], especially those related to sleep improvement. Xu [119] et al. found that ginsenoside Rg1(Rg1) was able to increase the total sleep, RNE sleep, and NRNE sleep duration in rats by modulating the noradrenergic system in the locus coeruleus and the serotonergic system in the dorsal nucleus of the midbrain. Furthermore, Rg1 has been demonstrated to reduce neuronal apoptosis, attenuate mitochondrial dysfunction induced by sleep deprivation, and improve memory through the AMPK-SIRT13 pathway [120]. Lee [121] et al. utilized a two-electrode voltage-clamp technique to investigate the impact of ginsenoside Rg3 (Rg3) from ginseng on GABAAR channel activity in Xenopus oocytes. The study revealed that Rg3 induced inward currents in Xenopus oocytes expressing GABAAR subunits (α1β1γ2), with this effect being selective for the expression of the γ2 subunit. Moreover, the application of GABAAR antagonists resulted in the inhibition of Rg3-induced inward currents. Shao [122] et al. found that ginsenoside Rg5 and Rk1, extracted from ginseng, could affect the GABA and 5-HT nervous systems by increasing the GABA/Glu ratio, up-regulating the expression of GABAA-R, GABAB-R, and 5-HT1A receptors, and thereby decreasing locomotor activity, improving the sleep quality index, shortening sleep latency, and lengthening mouse sleep time. In addition, certain scholars compared the sleep-improving activities of three protopanaxadiol-type (PD) ginsenosides (Rg3, Rk1, and Rg5) with those of protopanaxatriol-type (PT) ginsenosides (Rh1, Rk3, and Rh4). The findings revealed that both PD and PT ginsenosides possess sedative and hypnotic properties, mediated by the 5-HTergic and GABAergic systems. However, the activity of PT ginsenosides was found to be higher than that of PD ginsenosides at a high dose (96 mg·kg^−1^) [123].

Ginseng not only improves sleep via GABAergic and 5-HTergic activity, but also improves central fatigue, cognitive dysfunction, and hippocampal neuronal damage caused by sleep deprivation. In summary, ginseng is not only able to improve sleep but also the side effects caused by sleep deprivation, making it an excellent food for improving sleep.

### 4.5. Schisandra chinensis

*Schisandra chinensis Fructus (Schisandra)*, the dried ripe fruit of *Schisandra chinensis Turcz. Baill*., has been used in traditional Chinese medicine. It has many functions in the description of Chinese medicine and is often used in the treatment of insomnia [124]. It contains a variety of chemical components, including lignans, volatile oils, polysaccharides, and organic acids [125,126]. These chemical components play a role in the substance’s main bioactive components, which include anticancer, anti-inflammatory, sedative, hypnotic, and anxiolytic effects [127,128,129].

The crude extract of *Schisandra chinensis* has been shown to improve sleep. The supercritical carbon dioxide fluid extraction of *Schisandra chinensis* exerts sedative and hypnotic activity via the 5-HTergic and GABAergic systems, reduces sleep latency, and increases sleep duration in mice. This extraction also reverses caffeine-, PCPA-, and flumazenil-induced insomnia [124].

The lignans and Gomisin N in *Schisandra chinensis* are known to improve sleep. Schisantherin A (STA) and Schisantherin B (SchB) are the active lignans of *Schisandra chinensis* and represent some of the most abundant components of this plant. Both lignans have been shown to have sedative and hypnotic effects. In the course of the study, it was demonstrated that STA was capable of reducing autonomic activity and sleep latency whilst concomitantly increasing the number, time, and duration of sleep in mice. Furthermore, STA significantly increased GABA, decreased Glu in both the blood and brain, increased GAD in the brain, and up-regulated the expression of GABAARα1 and GABAARγ2 mRNA in rat brain tissue, thereby exerting a sedative and hypnotic effect [130]. SchB significantly reduced motor activity, improved the sleep quality index, increased sleep onset, increased sleep duration, shortened sleep latency, and prolonged sleep duration in mice. SchB can significantly increase the level of GABA and decrease the level of Glu in the peripheral blood of mice as well as in the cerebral cortex, hippocampus, and hypothalamus of rats, and SchB can up-regulate the expression of GABAARα1 and GABAARγ2 genes in the cerebral cortex, hippocampus, and hypothalamus [131]. Gomisin N is one of the major bioactive components in *Schisandra chinensis* fruit. In a seminal study, Zhang [132] et al. reported the effect of Gomisin N on the sedative and hypnotic activity of *Schisandra chinensis* and its mechanism for the first time. Gomisin N exerted a weak sedative effect on locomotor activity in normal mice, and in pentobarbital-treated mice, it produced a dose-dependent increase in sleep duration. It was also found to reverse PCPA- and caffeine-induced insomnia in a rodent model by altering the serotonin and GABAergic systems and to show synergistic effects with 5-HTP.

A combination of supplements including *Schisandra chinensis* has been shown to improve sleep. Zhumian Granules (including *Schisandra chinensis* and ZSS) can prolong sleep time and relieve PCPA-induced insomnia by increasing 5-HT and decreasing GABA, DA and NE [133]. AnMei Decoction (including ZSS, ginseng, *Schisandra chinensis*, etc.) not only treat sleep disorders, but also improve neuroinflammation, synaptic damage, and cognitive deficits associated with sleep deprivation by inhibiting the NLRP3/Caspase1 pathway and modulating the regulation of the BDNF/TrkB pathway [134].

In conclusion, both crude extracts and supplements of *Schisandra chinensis* are able to improve sleep through 5-HTergic and GABAergic effects. Therefore, it is a promising compound for product development.

### 4.6. Juglans regia

Walnuts (*Juglans regia*) have been utilized as a health food and folk medicine for millennia. Walnuts contain a variety of biologically active nutrients, including protein, monounsaturated fatty acids, polyunsaturated fatty acids, vitamins, melatonin, etc. [135,136]. A plethora of in vitro, in vivo, and clinical trials have demonstrated their capacity to exhibit various biological activities, including antioxidant, anticancer, anti-neuroinflammatory, and neuroprotective properties, cardiovascular disease risk reduction, accelerated diabetic wound healing, and sleep improvement [137,138,139,140,141,142,143].

At the human level, whole walnuts have been shown to improve sleep quality. A daily intake of 15 g of whole walnuts improved subjective sleep quality and cognitive performance in older men [144].

At the animal level, walnut peptides have been shown to improve sleep. By gavaging mice with walnut oligopeptides (WOPs), Zhu [27] et al. found that WOPs had no direct sleep effects and did not affect the rate of sleep onset but shortened the sleep latency, prolonged the duration of sleep, and increased the levels of GABA and 5-HT in the mouse brain. Walnut protein hydrolysate also alleviates the side effects of sleep deprivation, reverses sleep deprivation-induced memory deficits by reducing oxidative stress, normalizes the expression of catalase, glutathione peroxidase (GSH-px), and superoxide dismutase (SOD), and protects PC12 cells from glutamate-induced apoptosis [145].

At the animal level, the sleep-improving efficacy of 1,5-anhydrous-D-glucitol and 3-hydroxy-4-iminobutyric acid (HIBA) in walnut *Diaphragma juglandis fructus* is significant. The active ingredient responsible for improving sleep is also found in walnut *Diaphragma juglandis fructus*. The sleep-improving effects of 1,5-anhydro-D-glucitol and HIBA, isolated from walnut *Diaphragma juglandis fructus* were investigated. 1,5-anhydro-D-glucitol was found to significantly prolong sleep time (a 19.87% increase in natural sleep duration and an 87.00% increase in sodium pentobarbital-induced sleep duration); compound 6 not only caused the sleep duration of the mice to be prolonged by 61.49%, but also caused the sleep latency of the mice to be shortened by 24.62%, decreased the speed and duration of movement, and increased the duration of stay in the central region [146]. Among these, HIBA disrupts motor activity and prolongs sleep by modulating neurotransmitters (GABA, DA, etc.) in the mouse brain and serum. HIBA further alters basal ganglia metabolism (neurotransmitters, acylcarnitines, purine nucleosides, etc.) via the microbe–gut–brain axis, which affects the intestinal flora (*Flavonifractor, Parabacteroides, Bacteroides,* and *Lactobacillus*), which in turn modulates sleep quality in mice [147].

In summary, walnut, walnut *Diaphragma juglandis fructus,* and other parts of the fruit have sleep-improving activity, and walnut peptides are able to alleviate the side effects of sleep deprivation. Therefore, they are substances with potential for use in the manufacturing of sleep-improving products.

### 4.7. Others

There are also foods that have the function of improving sleep. These products cannot be categorized as above and are included here to be discussed separately according to the composition of the substances extracted from them.

Research on peptides: Wang [148] et al. found that a small peptide (TG7: YGNPWEK) isolated from the enzymatic digestion of *Pneumatophorus japonicus* bone was able to ameliorate abnormal changes in the locomotion of insomniac zebrafish by regulating circadian rhythms and visual functions and normalizing the distance, speed, and time of zebrafish locomotion. Lv [149] found that the enzymatic peptides from *Mauremys mutica* plastron could improve sleep in PCPA-induced insomniac mice by ameliorating neurotransmitter (5-HT, GABA, and DA) disorders and regulating the expression of the 5-HT1A receptor and GABAARα1 subunit.

Research on polyphenols: *Perilla frutescens* is used as a traditional folk sedative in Eastern countries. Rosmarinic acid, one of the main constituents of perilla, is a polyphenol capable of exerting sleep-improving effects through GABAergic pathways. It reduces the number of sleep/wake cycles and REM sleep in rodents, while increasing total sleep time and NREM sleep [150]. Kim [151] et al. found that apigenin was able to improve sleep by modulating GAD_65_ expression and increasing Cl^−^ inward flux.

Research on saponins: Cao [152] et al. found that tenuifolin from Radix Polygae, a saponin, was able to show sleep-enhancing effects in rodents, as analyzed by electroencephalographic (EEG) and electromyographic (EMG) analyses. *Gynostemma pentaphyllum* was found to prolong total sleep time by increasing NREM sleep and REM sleep. Additionally, saponins from *Gynostemma pentaphyllum* (Thunb.) Makino (*G. pentaphyllum*) were able to improve sleep by modulating GABAergic and 5-HTergic effects. The saponin fraction obtained from *G. pentaphyllum* was able to significantly reverse PCPA-induced insomnia symptoms. The saponin fraction GPMB was able to regulate the expression of 5-HT1A, 5-HT2A, and TNF-α, while GPMS even more significantly regulated the expression of GABAARα2, GABAARα3, GAD65/67, and IL-1β [153].

Research on oil: *Perilla frutescens* essential oil is able to improve the sleep onset rate, prolong sleep duration and multiple sleep latencies by increasing the levels of 5-HT and GABA in the hypothalamus and cerebral cortex, and increase the expression levels of GABAAα1 and GABAAα2 genes and proteins [154]. Valerian essential oil improves PCPA-induced insomnia by activating the 5-HTergic synaptic signaling pathway and increasing the expression of 5-HT and GABA [155].

Research on crude extracts: Traditionally, *Poria cocos* has been used to treat insomnia. Kim [156] et al. found that the ethanolic extract of *Poria cocos* was able to improve sleep duration and sleep latency in sleep-deprived mice by modifying the GABAergic system. Oh [157] et al. found that an aqueous extract of *Vaccinium bracteatum* Thunb had a sedative and hypnotic effect and was sufficient to reverse PCPA. It was able to reverse the loss of sleep time after PCPA modeling, activate 5-HT_1A_, and up-regulate the expression of GAD_65/67_, GABAAα5, β1, and β2.

The efficacy of these natural products is attributable to their ability to modulate the expression of neurotransmitters and receptors within the GABAergic and 5-HTergic systems, thereby enhancing sleep quality. This provides a natural alternative for the treatment of insomnia.

**Table 1:** Foods with sleep-improving effects and their mechanisms of action.

Categorisation	Ingredient	Known Chemical Components	Model	Time/Doses/ Duration	Results	Mechanism of Action	References
Peptide	Milk	YPVEPF, YFYPEL	Pentobarbital-induced sleeping tests	7 D, 5 mg/kg, p.o.	Increase sleep duration and sleep onset	Act at the benzodiazepine site of the GABAAR	[24]
Peptide	*Juglans regia*	Walnut oligopeptides	Pentobarbital-induced sleeping tests	30 D, 220, 440, 880 mg/kg, p.o.	Reduce sleep latency and increase sleep duration	Increase GABA and 5-HT levels in the brain	[27]
Peptide	Peptide	Small-molecule, soybean protein-derived peptide	Pentobarbital-induced sleeping tests	9 D, 0.65, 1.3. 2.60 g/kg, p.o.	Increase sleep duration	Increase the release of MT and up-regulate the MT1 and MT2 receptor activities	[72]
Peptide	Milk	A casein hydrolysate rich in the peptide YPVEPF	Unpredictable chronic mild stress procedure	14 D, 150 mg/kg, p.o.	Reduce sleep latency, increase sleep duration and sleep onset	Increase in GABA, 5-HT, GABAA, 5-HT_1A_ receptors, and BDNF and a decrease in IL-6 and NMDA receptors	[82]
Peptide	*Pneumatophorus japonicus* bone	TG7 (YGNPWEK)	200 lux light modeling insomnia model	24 h, 0.2 mg/mL, p.o	Decrease and normalize values for distance traveled, swimming speed, and activity time	Regulate visual function and improve circadian rhythms	[148]
Peptide	*Mauremys mutica* plastron	*Mauremys* mutica plastron peptides	Pentobarbital-induced sleeping tests and PCPA-induced insomnia model	12 D, 400 and 800 mg/kg, p.o.	Reduce sleep latency, increase sleep duration and sleep onset	Improve neurotransmitter system disorders and modulate 5-HT_1A_ and GABAARα1 subunit expression	[149]
Dairy products	Milk	Tryptic hydrolysate of αs1-casein	Pentobarbital-induced sleeping test and EEG	3 D, 30–240 or 150 mg/kg, p.o.	Reduce sleep latency and α wave, increase sleep duration and θ wave	Elevate protein expression of GABAAR β1 isoforms in the hypothalamus	[79]
Dairy products	Milk	*Lactobacillus brevis* DL1-11 fermented milk	Pentobarbital-induced sleeping tests	30 D, 8.83, 16, 67, and 33.33 mg/kg, p.o.	Reduce sleep latency, increase sleep duration	Regulate gut microbiota and increase SCFA levels	[84]
Saponin	Ginseng	Ginsenoside Rg1	EEG and EMG	3 D, 5, 10, and 20 mg/kg, p.o.	Increase the duration of total sleep, REM sleep, and NREM sleep	Regulate the noradrenergic system in the blue spot and the 5-HTergic system in the dorsal nucleus of the middle suture	[119]
Saponin	Ginseng	Ginsenoside Rg5/Rk1	Pentobarbital-induced sleeping tests	7 D, 30 and 60 mg/kg, p.o.	Reduce sleep latency and locomotor activity, increase sleep duration	Increase the GABA/Glu ratio, and up-regulate the expression of GABAAR, GABABR, and 5-HT_1A_	[122]
Saponin	Ginseng	Rare protopanaxadiol-type ginsenosides and protopanaxatriol-type ginsenosides	Pentobarbital-induced sleeping tests and insomnia model with caffeine	64 and 96 mg/kg, p.o.	Reduce sleep latency and locomotor activity, increase sleep duration	Activate 5-HTergic and GABAergic systems	[123]
Saponin	*Polygala tenuifolia*	Tenuifolin	Measurement of EEG and EMG	20, 40, and 80 mg/kg, p.o.	Increase NREM sleep, REM sleep, and sleep duration	Mediated by activation of the GABAergic system and/or inhibition of the noradrenergic system	[152]
Saponin	*Gynostemma pentaphyllum* (Thunb.) Makino (*G. pentaphyllum*)	Saponin-containing components	Sodium pentobarbital-induced sleeping	14 D, 150 mg/kg, p.o.	Reduce sleep latency and increase sleep duration	Increase the expression of 5-HT_1A_, 5-HT_2A_, GABAARα2, GABAARα3, GAD_65/67_, TNF-α, and IL-1β	[153]
Lignans	*Schisandra chinensis*	Schisantherin A	Pentobarbital-induced sleeping tests	7 D, 1,75, 3.5, and 7 mg/kg, p.o.	Decrease autonomic activity and sleep latency, increased sleep number and sleep duration in mice	Up-regulate the content of GABA and the expression of GABAAR and GAD, and reduce the content of Glu	[130]
Lignans	*Schisandra chinensis*	Schisandrin B	Pentobarbital-induced sleeping tests	7 D, 1.25, 2.5, and 5 mg/kg, p.o.	Decrease in the number of voluntary activities and sleep latency, increase in sleep rate and duration of sleep	Up-regulate the content of GABA and gene expression of GABAAα1 and GABA Rγ2, and reduce the content of Glu	[131]
Lignans	*Schisandra chinensis*	Gomisin N	Pentobarbital-induced sleeping test, caffeine-induced insomnia model, PCPA-induced insomnia model, and FLU-induced insomnia model	7 D, 5, 15, and 45 mg/kg, i.p.	Reduce sleep latency, increase sleep duration and sleep onset	Activate 5-HTergic and GABAergic systems	[132]
Lignans	*Ziziphus jujuba*	The terpenoids	Pentobarbital-induced sleeping tests	30 D, 0.5 and 1.5 g/kg, p.o.	Reduce sleep latency and increase sleep duration	Increase 5-HT and GABA levels, promote GABAAR expression, and decrease Glu and NE levels and IL-1β expression	[97]
Oil	*Moringa oleifera* Lam. Seed	*Moringa oleifera* Lam. Seed oil	Pentobarbital-induced sleeping tests	30 D, 0.5 and 1.5 g/kg, p.o.	Reduce sleep latency and increase sleep duration	Increase levels of GABA and Glu and protein expression of GAD_65_ and GABAARα1	[158]
Categorisation	Jinhua Hecha	Extracts	Pentobarbital-induced sleeping tests	44 D, 0.7, 1.6 and 2.3 g/kg, p.o.	Reduce spontaneous activity and sleep latency, increase sleep duration and fall asleep rate	Reduce Glu levels and maintain intestinal flora balance	[159]
Polyphenol	*Perilla frutescens*	Rosmarinic acid	Pentobarbital-induced sleeping tests and measurement of EEG	0.5, 1.0 and 2.0 mg/kg, p.o.	Decrease sleep latency, sleep/wake frequency, and REM sleep, increase sleep duration and NREM sleep	Increase the protein expression of GAD_65/67_, GABAARα3, α4, α5, β2, and γ3	[150]
Polyphenol	*Cirsium japonicum*	Apigenin	Pentobarbital-induced sleeping tests	12.5, 25 and 50 mg/kg, i.p.	Increase sleep duration and sleep onset	Increase the protein expression of GAD_65_ and Cl^−^ inward flux	[151]
Whole food	*Juglans regia*		Pentobarbital-induced sleeping tests	30 D, 220, 440, and 880 mg/kg, p.o.	Reduce sleep latency and increase sleep duration	Increase MT levels in the brain	[137]
Extraction	*Lactuca sativa*	Extracts	Vibration stress in *Drosophila Melanogaster* test, immobilization stress procedure in rats	9 D, 80 and 120 mg/kg, p.o.	Increase sleep duration, NREM sleep, and δ wave	Enhance expression of GABAAα2	[110]
Extraction	*Lactuca sativa*	Extracts	Pentobarbital-induced sleeping tests and insomnia model with caffeine	80, 100, 120, and 160 mg/kg, p.o	Increase sleep duration, NREM sleep, and δ wave	Bind to GABAAR	[111]
Extraction	*Lactuca sativa*	Extracts	Pentobarbital-induced sleeping tests and insomnia model with caffeine	21D, 50, 80, 100, and 150 mg/kg, p.o.	Increase sleep duration, NREM sleep, and δ wave	Enhance expression of GABAAR, GABAB1R, and 5-HT_1A_	[26]
Extraction	*Ziziphus jujuba* and Radix Polygalae	Extracts	Pentobarbital-induced sleeping tests and PCPA induced insomnia model	6D, 3.45, 6.9, and 13.8 mg/kg, p.o.	Reduce sleep latency, increase sleep duration and sleep onset	Increase GABA and 5-HT levels, decrease DA and NE levels, and regulate phenylalanine metabolism	[160]
Extraction	*Schisandra chinensis*	Extracts	Pentobarbital-induced sleeping test, caffeine-induced insomnia model, PCPA-induced insomnia model, and FLU-induced insomnia model	4D, 10, 25, 50, 100 and 200 mg/kg, p.o.	Reduce sleep latency and increase sleep duration	Activate 5-HTergic and GABAergic systems	[124]
Extraction	*Juglans regia*	3-hydroxy-4-iminobutyric acid	Pentobarbital-induced sleeping test	14 D, 0.5 g/kg, p.o.	Reduce sleep latency and activities, increase sleep duration	Increase GABA level, influence intestinal flora (*Flavonifractor, Parabacteroides, Bacteroides,* and *Lactobacillus*) by modulating basal ganglia metabolism (neurotransmitters, acylcarnitines, purine nucleosides, etc.)	[147]
Extraction	*Poria cocos*	Extracts	Pentobarbital-induced sleeping tests and caffeine-induced sleep disturbance animal model	10, 20, 40, 80, 160, and 320 mg/kg, p.o.	Reduce sleep latency, increase sleep duration and NREM sleep	Increase the protein expression of GABAA, GABAAB, and Cl^−^ inward flux	[156]
Extraction	*Vaccinium bracteatum Thunb.*	Extracts	Pentobarbital-induced sleeping tests and PCPA-induced insomnia model	29 D, 50 and 100 mg/kg, p.o.	Reduce sleep latency and activities, increase sleep duration	Activate 5-HT_1A_, increase the expression of GAD65/67, GABAAα5, β1, and β2	[157]
Extraction	Ashwagandha (*Withania somnifera* L. Dunal)	Extracts	Pentobarbital-induced sleeping test and caffeine-induced insomnia model and EEG	28 D, 100 mg/kg, p.o.	Increase sleep duration, NREM sleep, and δ wave	Increase the content of GABA and the expression of GABAAR and GABABR	[161]
Extraction	Leaf of Paullinia pinnata	Extracts	Phenobarbital sodium sleep-enhancing test	5 D, 100, 200, and 400 mg/kg, p.o.	Reduce sleep latency and increase sleep duration	Increase GABA levels in the brain	[162]

Notes: BDNF (brain-derived neurotrophic factor); EEG (electroencephalogram); EMG (electromyogram); GABA (gamma-aminobutyric acid); GABAAR (gamma-aminobutyric acid type A receptor); GABABR (gamma-aminobutyric acid type B receptor); GAD (glutamate decarboxylase); Glu (glutamic acid); IL-6 (interleukin-6); i.p. (intraperitoneal injection); NREM (non-rapid eye movement); PCPA (p-chlorophenylalanine); p.o. (oral administration); REM (rapid eye movement); SCFAs (short-chain fatty acids); 5-HT (5-Hydroxytryptamine); 5-HT1A (5-Hydroxytryptamine receptor 1A); 5-HT2A (5-Hydroxytryptamine receptor 2A); the lack of treatment time was not mentioned in the article.

## 5. Conclusions and Future Directions

The foods identified in the current study may improve sleep by modulating GABAergic and 5-hydroxytryptaminergic properties, providing a natural and relatively safe alternative for treating insomnia. Additionally, some sleep-improving substances not only enhance sleep quality but also alleviate insomnia-related side effects, such as memory loss, impaired gut barrier function, inflammation, and circadian rhythm disruption. Therefore, these foods are being used to develop very good options for sleep improvement products.

However, the present study has several limitations: (1) Most of the substances currently being investigated are derived from natural products that are already known for their sleep-improving, sedative, and anxiolytic effects. The review found that some substances with neuroprotective effects also have the potential to improve sleep, such as milk, *Ziziphus jujuba, Lactuca sativa*, ginseng, *Schisandra chinensis, Juglans regia*, etc., which have both neuroprotective and sleep-improving effects and can alleviate the side effects caused by insomnia. It is hoped that substances with this function can be screened in the future. (2) The mechanisms involved are limited. Currently, the efficacy and mechanisms of action of sleep-enhancing substances are primarily associated with GABA, 5-HT, and MT. Despite the recent increase in research on the microbiota–gut–brain axis, other mechanisms remain underexplored. Given the complexity of insomnia, which involves multiple factors, it is hoped that future studies will identify additional sleep-improving substances with novel mechanisms. The following mechanisms can be studied: the regulation of PGD2, adenosine, and calcineurin. (3) There are fewer pharmacokinetic studies on these ingredients. It is hoped that in the future, there will be more pharmacokinetic studies on related substances which will help to understand the mechanism of action of this ingredient and to evaluate the safety and efficacy of sleep aid products. (4) There are no in vitro and direct screening indicators. Currently, most screening of active ingredients for sleep improvement relies on animal experiments, while in vitro screening is conducted indirectly through methods such as antioxidant properties and molecular docking analysis. There is a need for direct and effective in vitro screening methods. Future work should focus on developing in vitro indices or cellular models to screen for active ingredients.

## Data Availability

No new data were created or analyzed in this study. Data sharing is not applicable to this article.
